# The effectiveness and economic evidence of organizational and management interventions to promote mental wellbeing and resilience in elderly care workers and informal caregivers – a systematic review

**DOI:** 10.1186/s12913-025-13372-7

**Published:** 2025-10-10

**Authors:** Anna-Kaisa Vartiainen, Daniel Adrian Lungu, Elisa Rissanen, Luca Pirrotta, Luca Scopis, Tanja Schroeder, Maren Sogstad, Kristian R. Odberg, Arne Hole, Juana María Delgado-Saborit, Mari Lahti, Cecilie Haraldseid-Driftland, Nicola Belle, Louise Ellis, Siri Wiig, Eila Kankaanpää

**Affiliations:** 1 Department of Health and Social Management, University of Eastern Finland, Kuopio, Finland; 2 SHARE Centre for Resilience in Healthcare, Faculty of Health Sciences, University of Stavanger, Stavanger, Norway; 3 Sant’ Anna School of Advanced Studies, Pisa, Italy; 4 Australian Institute of Health Innovation, Macquarie University, Sydney, Australia; 5 Department of Health Sciences, Norwegian University of Science and Technology, Gjøvik, Norway; 6 Jaume I University, Castellón de la Plana, Spain; 7 Health and Well-being, Turku University of Applied Sciences, Turku, Finland

**Keywords:** Resilience, Mental wellbeing, Effectiveness, Cost-effectiveness, Elderly care worker, Informal caregiver, Management, Long-term care

## Abstract

**Background:**

The increasing number of older adults presents significant challenges for healthcare systems. Elderly care workers, leaders and informal caregivers are facing challenges impacting their mental wellbeing and resilience, necessitating targeted organizational and management interventions. This systematic review aims to evaluate the effectiveness and economic evidence of organizational and management interventions designed to promote mental wellbeing and resilience among elderly care workers, leaders, and informal caregivers.

**Methods:**

A systematic search was conducted on the CINAHL, PsycINFO, Scopus, Web of Science, and PubMed databases. We included all management and organizational intervention studies focusing on elderly care workers and informal caregivers, and aiming to promote their mental wellbeing and resilience. We included randomized controlled trials and observational studies with both intervention and comparison groups, and studies with an economic outcome that combines costs and consequences. The methodological quality of the studies was assessed using Cochrane risk of bias tools and Consensus Health Economic Criteria.

**Results:**

The final searches yielded altogether 5,700 articles, from which 15 studies were included in this review. Of those, seven explored elderly care workers interventions and eight explored informal caregiver interventions. Most interventions were targeted at individual workers rather than organizational practices. Among the elderly care worker interventions summarized, only a few demonstrated a positive effect on promoting mental wellbeing. Notably, mindfulness, breathing exercises, and Acceptance and Commitment Therapy (ACT) were found to be beneficial for promoting wellbeing, although there are limitations to consider when interpreting the results. In contrast, a higher proportion of informal carer interventions showed positive outcomes related to mental wellbeing. Effective strategies included support and counselling, practical skills training, awareness and knowledge improvement, individual coping therapy, and computer-assisted care management protocol. Only one economic evaluation study was found. The psychological coping intervention for caregivers (START) was cost-effective.

**Conclusion:**

Enhancing the coping of informal caregivers with tailored interventions can promote their mental wellbeing and resilience. This review indicates that there is a notable scarcity of system-level interventions or comprehensive research on such interventions. While it is relatively straightforward to identify problems within the caregiving field, finding evidence-based solutions remains challenging.

**Trial registration:**

PROSPERO 2024, CRD42024551372.

**Supplementary Information:**

The online version contains supplementary material available at 10.1186/s12913-025-13372-7.

## Background

According to the World Health Organization, by 2030 one in six people in the world will be aged 60 years or over, and the number of people aged 80 years or older is expected to triple by 2050 [1]. The increasing number of older adults, particularly those aged 85 and above, who are most likely to need health and care services, presents significant challenges for healthcare systems [1, 2]. As life expectancy increases, the complexity of care needs also grows, often involving chronic conditions, cognitive impairments, and end-of-life care [3, 4].

The provision of elderly care is a critical component of healthcare systems worldwide, reflecting the growing demographic of aging populations. This includes a diverse range of services aimed at supporting the physical, emotional, and social needs of older adults. Elderly care includes assisted living, adult daycare, long-term care, nursing homes, hospice care, and home care, emphasizing the social and personal requirements of senior citizens who wish to age with dignity while needing assistance with daily activities and healthcare. This demographic shift increases the complexity of care needs and places greater demands on both formal and informal caregiving systems. Informal caregivers, typically family members or friends, provide essential care without formal training or support, often balancing caregiving with other responsibilities [5].

Meeting the growing demand for care requires coordinated efforts from formal care systems and informal networks, yet many countries face resource limitations, workforce shortages, and systemic inefficiencies [6]. These pressures can contribute to increased stress and emotional burden for those providing care [7].

Elderly care workers - including nurses, healthcare assistants, and support staff - who are often on the front lines of care delivery, are exposed to high levels of stress and emotional labour [8]. Studies have shown that care workers frequently experience burnout, emotional exhaustion, and high levels of job-related stress [9–12]. Prolonged exposure to these stressors can lead to absenteeism, reduced job performance, and ultimately, workforce attrition [13].

Informal caregivers often report feelings of isolation, anxiety, and depression, highlighting the need for interventions that support their mental wellbeing and resilience [14, 15]. Estimates suggest that between 40 and 70% of informal caregivers experience symptoms of depression, and many rate their caregiving experience as highly stressful [16]. The dual role of managing their own lives while providing care can lead to high levels of caregiver burden and burnout [17].

These work demands and working conditions in elderly care and informal care necessitate special attention to promote mental health and wellbeing at work. As defined by the World Health Organization, mental health is “a state of mental well-being that enables people to cope with the stresses of life, realize their abilities, learn well and work well, and contribute to their community” [18]. Mental wellbeing in elderly care and informal care refers to a positive state of mental health, including the ability to manage stress, maintain meaningful relationships, and function effectively at work and in daily life.

The mental wellbeing of workers and informal caregivers is crucial not only to their own health but also to the quality of care they provide. The quality of care is directly impacted by the mental wellbeing of caregivers, with higher levels of burnout linked to lower patient satisfaction and increased risk of errors [19, 20].

The costs related to poor mental health are considerable. The main costs accrue from lost production, reduced performance due to psychosocial problems and use of health and social care services [21]. In 2013, the total cost of work-related depression alone in the EU-27 was estimated to be €620 billion per year. Employers face most of the costs due to absenteeism and presenteeism (€270 billion). The economy as a whole bears a share in terms of lost output (€240 billion), services provided in health care (treatment costs €60 billion) and in social welfare systems (disability benefits €40 billion) [22].

Concrete changes in the workplace can promote the mental wellbeing of health care workers. Interventions including job and task modification, flexible work scheduling or changes in physical environment have shown evidence to improving mental wellbeing, especially decreasing burnout [23]. The evidence of individuals targeted mental health promoting interventions has been inconsistent [24]. Previous reviews of interventions targeting informal caregivers have shown mixed effects and limited long-term sustainability [25]. However, these caregivers often receive support via organizational actors, such as community health systems or municipal services, justifying their inclusion in reviews focused on system-level interventions.

Research has shed light on the importance of resilience in healthcare settings [26]. It widens the scope of mental health; resilience is not merely an individual trait but also a characteristic of systems and organizations [27]. Resilience, defined as organizational capacity to adapt challenges and changes at different system levels, to maintain high quality care, is particularly important in this context [27]. Resilient teams demonstrate flexibility, maintain care quality during disruptions, and innovate in response to changing needs. Resilient work units have adaptive capacity in the face of challenges [28] and are able to innovate their work practices during times of disruption, such as COVID-19 [29–31]. There is a need for organizational and management interventions that not only address immediate stressors but also build long-term resilience among care providers [24].

Leaders in elderly care settings play a crucial role as change agents in creating a supportive work environment and implementing policies that promote mental wellbeing and resilience among staff. Effective leadership is associated with better job satisfaction, reduced turnover, and improved mental health outcomes for care workers [32, 33]. Leadership style and organizational culture significantly influence the resilience and mental health of the workforce [34].

Economic evaluation helps decision makers choose the best ways to use resources efficiently for treatments, care services, or prevention [35]. It involves comparing the costs obtained and consequences achieved by at least two different interventions or comparing an intervention to “no action”. An economic evaluation may be integrated with a trial, or data on costs and effects from various sources can be combined with decision modelling [36]. Despite the relevance of cost-effectiveness, few reviews have examined the economic dimensions of caregiver support interventions alongside their effectiveness [37, 38].

The need for effective interventions to promote wellbeing is essential. Improving mental wellbeing and resilience among caregivers is not only a health priority but also an economic imperative. This systematic review aimed to evaluate the effectiveness and economic evidence of organizational and management interventions designed to promote mental wellbeing and resilience among all elderly care workers, and informal caregivers. The review investigates a wide range of potential interventions, including training programmes, support groups, policy changes, and technological innovations. We include informal caregivers in our scope because many support interventions—such as respite care, education, or peer support—are implemented through health or social care systems, making them relevant in an organizational context.

By focusing on the effectiveness and economic evaluation of these interventions, this review seeks to inform policy and practice, ultimately enhancing the support available to these key stakeholders.

## Methods

The protocol of the review is registered in PROSPERO (registration number CRD42024551372) [39]. A systematic literature review was conducted to gather and synthetize empirical evidence from published scientific sources. As applicable, this systematic literature review is reported according to the Preferred Reporting Items for Systematic Review and Meta-Analyses (PRISMA) [40]. There were no changes to the protocol.

### Literature search

The search string was created using the PICOs method, which covers population, intervention, comparison, outcome and study design. We searched the literature concerning elderly care workers and informal caregivers. We included all individual, management and organizational interventions, that promoted the mental wellbeing or resilience of workers, leaders and informal caregivers defined as positive concepts of mental health such as quality of life or psychological wellbeing. For effectiveness studies, there had to be at least one outcome measuring the mental wellbeing or resilience of the workers. For study design, we included randomized controlled trials (RCT), cluster randomized controlled trials (cRCT) and observational studies with both intervention and comparison groups. We accepted all economic evaluations: any economic evaluation outcome (i.e. incremental cost-effectiveness ratio, cost-benefit ratio, benefit-cost ratio, net present value). More specific inclusion and exclusion criteria can be found in Table 1.

**Table 1:** Inclusion and exclusion criteria using PICOs

**Study characteristic**	**Inclusion criteria**	**Exclusion criteria**
Population	Elderly care workers and informal caregivers	Residents/Elderly people
Intervention	Organizational or management interventions, promoting mental wellbeing/mental health and resilience (defined as positive concepts of mental health such as quality of life, general wellbeing, psychological wellbeing)	Studies on workers’ perceptions or attitudes of interventions generally Interventions to reduce mental health-related attitudes/stigma among workers Interventions (e.g., clinical) treating mental health condition
Comparator(s)	Intervention has (any reported) comparison group	
Outcome	For effectiveness studies; at least one measure of mental health, mental wellbeing or resilience (such as QALY, CD-RISC, general wellbeing)	Studies with measures of work environment factors/ organizational measures only
	For economic evaluation; any reported outcome (i.e. incremental cost-effectiveness ratio (ICER), cost-benefit ratio, benefit-cost ratio, net present value).	
Study Design	Effectiveness studies	
	Economic evaluations: cost-effectiveness analysis, cost-utility analysis, cost-benefit analysis and cost-minimization analysis and cost-consequence analysis.	
	Accepted study designs are experimental (RCT, cRCT) and observational studies.	
	Modelling studies are included if they rely on empirical effectiveness data	
Publication	Published in a peer-reviewed journal	Reviews, study protocols, editorials, letters to the editor, commentaries, theses or dissertations
	Published since 2000	Grey literature
	Language is English	

Preliminary searches were conducted to assess the relevance of keywords and to identify possible missing words. A university librarian was consulted both for the preliminary search and for the final search. The final search was conducted on 31 st of May 2024 across several databases: CINAHL (EBSCO), PsycINFO (EBSCO), Scopus, Web of Science (Web of Science TM Core Collection), and PubMed (MEDLINE). Search was limited to publications from year 2000 onwards. Words related to mental wellbeing, resilience, intervention, elderly care, management and organizational, effectiveness and economic evaluation were combined to conduct the search. The search strategy can be found in Additional file 1.

### Study selection

After completing the search, references were imported to the Covidence software platform and duplicates removed. First, all review members (AKV, ER, DL, TS, LS, LP, KO, MS, AH) went independently through a random sample of studies (5% of the final search result, *n* = 125) and all together discussed the inclusion and exclusion criteria to ensure consistent practice. Then, screening of the studies (title and abstract) was done in duplicate among review members. After members had completed the screening, a meeting was organized for discussion and for decision making about conflict cases. Conflicts were handled through structured discussion against eligibility criteria among review members to reach agreement. At the end of each conflict case discussion, the lead author provided a summary of proposed solution based on the discussion and others confirmed agreement. In the full text screening phase, we followed the same process and after screening was completed, possible conflicts were handled and decisions made in an online meeting.

We had 29 random pairs for screening abstracts and titles and 19 random pairs for full text screening. The consistency of decisions of the review members was assessed by the Kappa statistic [41]. Weighted average Cohen’s kappa for title/abstract screening was 0.64 (substantial agreement) and for full text screening, Cohen’s Kappa was 0.58 (moderate agreement) between reviewers.

### Data extraction

Data from the studies were independently extracted by two reviewers (AKV and EK) and assessed for accuracy by other review members (DL, TS, LP, LS, KO, MS) using a predesigned Microsoft Excel sheet. If the information in the article was not accurate enough, then the authors of the study were contacted by the lead author (AKV). If reviewers observed failures in extracted data accuracy, they informed the lead author (AKV). The extracted data consisted of: details about the study and study setting; details of the intervention and control conditions; the study population, participant demographics, baseline characteristics, recruitment and study completion rates; the study methodology; primary effectiveness outcome and secondary outcomes, follow-up time, and effectiveness results; costs (if economic evaluation), the year of the costs, the perspective of the analysis and the discount rate; the primary outcomes of economic evaluation; the methods and results of sensitivity analysis and the authors’ conclusion about the results of effectiveness and economic evaluation.

### Methodological quality assessment

The methodological quality of the studies was assessed in duplicate by independent reviewer-pairs (AKV & EK, DL & TS, LS & LP, KO & MS). If necessary, reviewers discussed quality assessment tools and their interpretation with their review-pair before and during assessments to align their understanding and interpretation of the tool. After completing the assessments, pairs compared their results and any disagreements that arose between the reviewers were resolved through discussion, or with a third reviewer. Interrater reliability of quality assessments was not measured. We used the Cochrane Rob 2.0 tool for the RCT studies [42] and Risk Of Bias In Non-Randomized Studies (ROBINS-I) [43] for observational studies to assess the risk of bias. For the economic evaluation studies, the Consensus on Health Economic Criteria (CHEC) list was used [44]. Studies were not excluded based on the quality assessment.

### Data synthesis

The review and its findings are reported in accordance with the PRISMA guidelines [40] as applicable. Originally, different outcome measures were planned to be transformed into standardized effectiveness measures such as Cohen’s d. However, we were not able to complete this due to the number of different outcomes used in the studies. Thus, extracted data was summarized and described to answer the review question using narrative and numeric manners in the text and tables.

Studies concerning elderly care workers and informal caregivers are separately reported throughout the results section. Informal caregivers differ from health care workers, since they have no employment contract with any organization, so it is relevant to examine them as their own group. We grouped the interventions if possible and report the description and effectiveness results by these groups and combine the quality assessment with the effectiveness results. This enables to make conclusions for practice and show quality gaps in research.

## Results

**Fig. 1:**
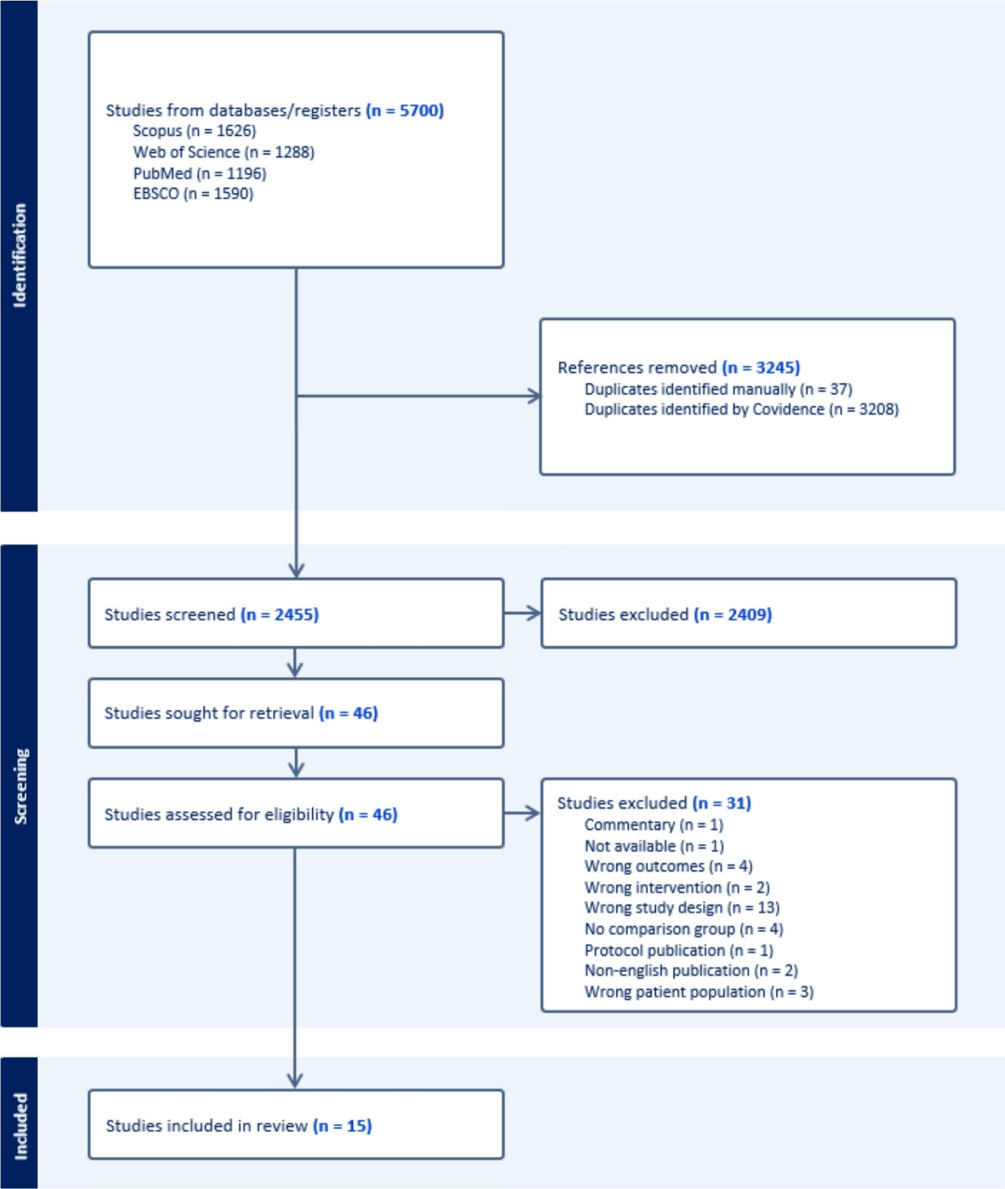
PRISMA diagram

The final searches yielded altogether 5,700 articles (Fig. 1). After duplicate removal, there were 2,455 articles. Of these, 2,409 articles were excluded based on the title and abstract. Full text was investigated from 46 articles and a further 31 articles were excluded. The final number of articles included in the review was 15 (two articles from the same study). Of these 15 articles, seven studied elderly care interventions [45–51] and eight informal caregiver interventions [52–59].

### Study characteristics (elderly care workers)

Research concerning elderly care workers and leaders has received more attention in recent years, since five out of seven studies were published after 2020. Four studies were conducted in the US and Canada, and the rest in Europe (UK, Italy, the Netherlands). Two of the studies were randomized controlled trials (RCT), and three were experimental/quasi-experimental. One study used a mixed methods study design, and one categorized their study as a feasibility study. Most of the study participants were workers (e.g. nurses) rather than managers or leaders. Study characteristics can be found in Table 2 and a more detailed description of the content of the interventions for workers in the section ‘Overview of the elderly care worker interventions’.

**Table 2:** Characteristics of the included studies

Author, year, country	Study population	Study design	Intervention, *n*	Comparison, *n*	Length of intervention and follow-up
**Elderly care workers**					
DeGraves et al. 2023, Canada [49]	Healthcare workers in long-term care (care aides, registered nurses, licensed practical nurses, or managers).	Pre–post intervention study	Basic Coherent Breathing, one breathing session daily, five to seven times a week, gradually increasing from 2 to 10 min per session, *n* = 112	Comprehensive Breathing (including device), same frequency as basic group, *n* = 142	8-week intervention
Hansell et al. 2023, USA [50]	Home care aides.	Feasibility study	Mindful awareness practices (MAPs), weekly in-person 120-minute classes, *n* = 30	Korean style Tai Chi, weekly in-person 120-minute classes, *n* = 25	6-week intervention, 3-month follow-up
Kloos et al. 2019, The Netherlands [46]	Nursing staff of nursing homes.	cRCT	Positive psychology intervention, one lesson per week individually at home, N/A duration of the lesson, *n* = 79	No intervention, *n* = 49	8–12-week intervention
McGilton et al. 2023, Canada [51]	Staff in long term care homes.	Pre-experimental static-group comparison design	Huddles, workplace discussions facilitated by nurse practitioner twice a week for day and night shifts, (15 min each), *n* = 20	Non-attendees, *n* = 22	15-week intervention
O’Brien et al. 2019, USA [45]	Nurses and nurse aides working in long-term care settings.	Experimental randomized study	Acceptance and Commitment Therapy (ACT), A two-session (2.5 h per session) group-based intervention, *n* = 37	Waiting list (after 1 month) *n* = 34	1 week intervention, 4-week follow-up
Riello et al. 2021, Italy [47]	Nursing or care home workers.	RCT	Self-help audio-visual tool (SH+) psychological intervention, individually at chosen time, N/A for duration of each session, *n* = 119	Alternative activity (compassionate reading), individually at chosen time, N/A for duration of each session *n* = 119	5-week intervention, 1 and 14-week follow-up
Schoultz et al. 2022, UK [48]	Nurses and carers in a private care home.	Sequential mixed methods design	Psychological First Aid (PFA) training, a free-to-access online training, N/A for duration of the training, *n* = 37	Not completed PFA training, *n* = 351	N/A
**Informal caregivers**					
Araújo et al. 2018, Portugal [53]	Informal caregivers for stroke patients	Quasi-experimental design	InCare counselling intervention, three home sessions ranged 45–90 min according to needs of each caregiver over three months and telephone counselling, *n* = 85	The usual type of care delivered in healthcare units, *n* = 89	3 months intervention
Dias et al. 2008, India [52]	Informal caregivers for dementia patients	RCT	Home based support intervention, visits carried out at least once a fortnight for six months, N/A for duration of the visits, *n* = 41	Waiting list control (after 6 months), *n* = 40	6 months intervention
Ducharme et al. 2005a & 2005b, Canada [57, 58]	Adult- daughter primary caregivers for dementia patients living in the residential centre	RCT	’Taking care of myself’ support intervention, ten 90-minute weekly sessions for groups of six to eight caregivers, *n* = 45	Alternative programme provided by a Quebec Alzheimer Society (AG), 10 weekly sessions, *n* = 51 Control group with no programme (CG), *n* = 41	2005a: 10 weeks intervention 2005b: 3 months follow-up
Duncan Davis et al. 2011, USA [56]	Caregivers who had recently placed a family member with dementia into a nursing home	RCT	FITT-NH counselling intervention, 10 telephone contacts over 3 months (Iinitial contacts 60 min and follow-up and termination calls 35–45 min) *n* = 27	Non-contact control condition, *n* = 26	3 months intervention
Haley et al. 2008, USA [54]	Informal caregivers for elderly with Alzheimer’s disease	RCT	NYUCI counselling intervention, during the first 4 months two individual and four family counseling sessions, weekly support group for AD caregivers, and “ad hoc” counseling by phone or face-to-face, *n* = 122	Treatment as usual, *n* = 132	N/A for intervention, up to 6 years follow-up
Livingston et al. 2014, UK [55]	Family carers of patients with dementia	RCT	START (STrAtegies for RelaTives) coping intervention, eight manual-based sessions, N/A for duration of each session, *n* = 173	Treatment as usual, *n* = 87	Short term follow-up: 4 and 8 months, and long term follow-up: 12 to 24 months
Montgomery et al. 2011, USA [59]	Care managers and caregivers	RCT	TCARE care management protocol, managers *n* = 23 carers *n* = 143	Treatment as usual, managers *n* = 29 carers *n* = 123	3, 6 and 9 months follow-up

### Study characteristics (informal caregivers)

Of the 15 articles, eight explored informal caregiver interventions. Studies concerning informal caregivers were published around year 2010 and the most recent article was from year 2018. Five studies were conducted in US and Canada, two in Europe (UK, Portugal) and one in India. Seven studies were RCT studies, and one was a quasi-experimental study. The study characteristics can be found in Table 2 and more detailed description of the content of interventions in section ‘Overview of the informal caregiver interventions’.

### Overview of the elderly care worker interventions

The interventions differed substantially across studies. Six interventions out of seven were targeted at individual workers rather than organizational practices. Altogether four interventions were based on psychology and offered skill-training or focused on raising awareness and knowledge of mental health and wellbeing and coping skills [45–48]. One of them was group-based therapy with exercises [45], three were individually completed psychoeducation and training courses; two was directed at oneself [46, 47], and one to support others [48]. Two interventions focused on physical exercises [49, 50] and one intervention assessed organizational practices through huddles [51].

Psychological interventions varied in mode of delivery and comparison groups. The study by O´Brien et al. [45] introduced a group-based Acceptance and Commitment Therapy (ACT) that involved two 2.5-hour sessions focusing on acceptance, mindfulness, psychological flexibility, willingness to experience discomfort, present-moment focus, self-as-context, values identification, and values-congruent committed action. ACT therapy group was compared to waiting list controls [45].

Other psychological interventions were completed individually. Kloos et al. [46] studied an online gamified positive psychology intervention, based on the existing multicomponent ‘This is Your Life’ programme. The intervention lasted approximately 8 weeks (1 lesson per week), but participants had the opportunity to use materials for 12 weeks. The intervention consisted of psychoeducation and exercises on six topics of well-being: positive emotions, discovering and using strengths, optimism, self-compassion, resilience, and positive relations. Exercises were evidence-based positive psychology exercises (e.g. ‘the three good things’ or ‘imagine your best possible self’). The intervention aimed to improve general wellbeing, job satisfaction, and work engagement and was compared to no intervention controls [46]. The third psychological intervention studied by Riello et al. [47] was individually used self-help audio-visual tool (SH+) developed by the WHO. Participants performed the intervention according to their own schedule over 20 weeks. The study compared the SH + intervention to another activity, compassionate reading [47]. The fourth psychological intervention was the Psychological First Aid (PFA) programme, a free-to-access online WHO-developed brief training course studied by Schoultz et al. [48]. Three main principles of PFA are: a) to look (for safety or for who needs help); b) to listen (to those that are in distress); c) and to liaise (to further support). The study compared the intervention to participants, who did not complete the PFA [48].

Two interventions targeting physical and mental relaxation were found. DeGraves et al. [49] studied the Basic breathing intervention. The intervention included education sessions with optional virtual support sessions and coherent breathing over an 8-week period. Basic breathing was compared to another intervention, Comprehensive breathing, which included a device in addition to the basic breathing intervention [49]. Hansell et al. [50] examined Mindful Awareness Practices (MAPs) compared to Korean Style Tai Chi- interventions. Both MAPs and Korean-style Tai Chi involved weekly 120-minute sessions with a certified instructor. Both interventions lasted for six weeks and had a three-month follow-up [50].

Huddles studied by McGilton et al. [51] was the only organizational or managerial intervention. Huddles were facilitated by a nurse practitioner and huddle approach aimed to support cultural changes in the workplace through staff discussions. Huddles were conducted twice a week for day and night shifts over 15 weeks (48 huddles, 15 min per huddle). The facilitator nurse underwent training and received a toolkit equipped with resources to efficiently conduct huddles. The toolkit included an overview of the huddle structure, scripted guidelines for delivering structured huddles, as well as documentation and reflection sheets. The huddles aimed to engage staff from various disciplines, fostering collaboration to identify and address improvement opportunities raised by staff, ensuring a prompt and effective response [51].

### Overview of the informal caregiver interventions

Seven out of eight interventions targeted at informal caregivers concentrated on support and counselling and were delivered in different ways. Informal caregivers are rarely employers of any organization, and accordingly, there were no explicit organizational or management interventions. Two of the interventions offered support and knowledge through home visits [52, 53], two interventions provided support for coping through individual therapy sessions or involving both individual and family sessions [54, 55]. One offered counselling and support intervention provided by telephone [56], and one offered support through discussions and exercises in group sessions [57, 58]. One study investigated a care management protocol [59].

The Home-Based intervention studied by Dias et al. [52] was designed to improve dementia awareness and provide emotional support to the caregiver. The intervention involved flexible home-care visits tailored to the needs of the caregiver and family. Visits were carried out at least once a fortnight for six months and the intervention was compared to waiting list controls [52]. Araújo et al. [53] studied the InCare programme which provided home and telephone counselling and support for stroke rehabilitation from a nurse and was tailored to the caregiver’s needs. The intervention was conducted over three home sessions, each lasting between 45 and 90 min, offering practical skills and decision-making assistance. In addition, the programme provided telephone counselling. The InCare intervention lasted over three months and was compared to usual type of care delivered [53].

Haley et al. [54] studied The New York University Caregiver intervention (NYUCI), which offered a comprehensive support approach including two individual and four family counselling sessions delivered by trained professional counsellors. In addition, the intervention offered a weekly local support group for Alzheimer’s disease (AD) caregivers, and ‘ad hoc’ counselling by phone or face-to-face. Contents were tailored, for example, to provide education and resource information about AD, and coping with different situations [54]. Another coping intervention studied by Livingston et al. [55], the STrategies for RelaTives (START) programme offered an eight-session manual-based individual therapy and was delivered by supervised psychology graduates. Both interventions described were compared to treatment as usual [54, 55].

Counselling provided by telephone only was offered in the programme studied by Duncan Davis et al. [56]. Intervention was called the Telephone Tracking-Nursing Home (FITT-NH) intervention, and it consisted of 10 telephone sessions over three months providing emotional support, directing caregivers to appropriate resources, and teaching caregivers’ strategies to cope with ongoing problems during the transition to institutional placement. Intervention was compared to non-contact controls [56].

Ducharme et al. [57, 58] studied group-based support intervention called the ‘Taking Care of Myself’ programme. It aimed at the empowerment of caregivers by raising awareness of their own capacities [57, 58]. The intervention also helped the caregiver in applying a reframing coping strategy for dealing with painful emotions. The intervention consisted of ten weekly 90-minute group sessions (of six to eight caregivers), involving active participation in discussions and role-play exercises and written exercises. It was compared to another intervention and to no intervention. The comparison intervention was developed independently and provided by the Quebec Alzheimer Society and consisted of 10 weekly sessions [57, 58].

Montgomery et al. [59] studied The Tailored Caregiver Assessment and Referral protocol (TCARE), a computer-assisted manualized care management protocol, which is specifically designed for care managers working with caregivers. TCARE aimed to empower caregivers to make informed decisions by providing them with information about the care context, their own strengths and needs, and the resources available to address their needs. The intervention was compared to treatment as usual group [59].

### Effectiveness and economic evaluation results

#### Elderly care workers

Research designs used in the studies were heterogeneous, and risk of bias were moderate/some concerns in four studies [45, 47, 49, 51] and serious/high in three studies [46, 48, 50]. The main results are summarized in Table 3 and detailed risk of bias assessments in Additional file 2.

The results of psychological interventions to promote wellbeing were mixed based on four studies [45–48]. An experimental randomized trial by O´Brien et al. [45] assessed the effectiveness of group-based ACT for nurses and nurse aides. Within group analysis showed that participants in the treatment group reported a significant reduction in mental health symptoms and no significant changes were observed in the waiting list control group at one month’s follow-up. However, the study reported between group comparisons at one month follow-up, showing that the treatment group had only marginally significant fewer mental health symptoms relative to the waiting list control group (F (1, 52) = 3.20, *p* = 0.08). The result was associated with a small/medium effect size (**η²** = 0.06). The ACT intervention was associated with fewer work-related injuries and improved wellbeing, demonstrating its potential to enhance mental health among long-term care staff [45], but had some concerns regarding the risk of bias assessment. The study was generally well conducted but had some limitations concerning unclear handling of missing data and lack of pre-defined analysis plan.

Other psychological interventions were delivered individually using psychoeducation materials [46–48]. The online multicomponent positive psychology intervention studied by Kloos et al. [46] was not effective in promoting general wellbeing and work engagement compared to a no intervention control group but found significant condition and time interaction effect sustaining job satisfaction whereas it decreased in the control group (F = 4.54, *p* = 0.04, d = 0.10) [46]. Risk of bias was assessed as high due to concerns in multiple dimensions, such as bias in randomization process, and reporting the results. The study by Riello et al. [47] evaluated self-help psychological intervention, compared it to another alternative intervention, compassionate reading. Using a one-tailed χ²-test, the study results showed no significant difference in the rate of self-reported symptoms between the intervention group and the alternative intervention group across multiple follow-up points. At the one-week post-intervention follow-up point, mild symptoms were reported in 74.12% of intervention participants versus 76.47% in the alternative intervention group (χ² = 0.127, *p* = 0.361). Outcomes for resilience, wellbeing, and perceived stress also did not meet significance [47]. The study was generally well conducted but had some concerns in bias due to missing outcome data (dropout rate exceeded 25%). The study by Schoultz et al. [48] examining the impact of PFA training found significant differences between the PFA and non-PFA groups in terms of the specific features of coping, safety, calmness, and hopefulness, with those in the PFA group responding more favourably. The PFA group showed enhanced coping scores with small effect size (d = 0.27) compared to those who reported no completion of the training. However, no significant differences were observed more broadly in stress, connectedness, or accomplishment. Besides the low effect size, the number of participants who completed PFA training was very low and the risk of bias was assessed as high, indicating that the results should be taken with caution [48]. Study design, self-selection bias, and reliance on self-reported data introduced risks in study quality.

Based on the results of two studies, relaxation interventions can have a positive effect on the mental health of elderly care workers [49, 50]. In a study by DeGraves et al. [49] evaluating the impact of two slightly different coherent breathing interventions (group with device and no device), significant improvements were observed across several mental health and resilience measures among long-term care staff in both groups. Utilizing a mixed-effects linear regression model, the researchers found statistically significant reductions in perceived stress (PSS-10, b = −2.5, *p* < 0.001), psychological distress (PHQ-4, b = −0.9, *p* < 0.001), anxiety (PHQ-4, b = −0.5, *p* < 0.001), and depression (PHQ-4, b = −0.4, *p* < 0.001). No differences were found between Basic Coherent Breathing interventions with or without a device with respect to these measures. However, significant time-by-group differences were observed for insomnia (ISI, b = −1.3, *p* = 0.04, 95% CI = −2.5, −0.03) and burnout (ProQoL-BO, b = −0.7, *p* = 0.01, 95% CI = −1.3, −0.2), favouring the Basic comprehensive breathing group without a device [49]. In addition to breathing, mindfulness practices seem to have positive effects. In a study by Hansell et al. [50] Mindful awareness practices (MAPs) and Korean style Tai Chi among home care aides were compared using mixed models’ regression, and both interventions showed significant improvements in anxiety, depression, positive affect, perceived stress, insomnia, sleep quality, and mindfulness at six weeks’ time point. However, only the MAPs group sustained positive impacts, with continued progress in reducing negative affect at three month follow up. MAPs were also rated as more practical for daily life implementation compared to Tai Chi [50]. Notably, these two previous studies [49, 50] did not have ‘no intervention’ control group, which limits the reliability of conclusions about the effectiveness of the interventions. In addition, risk of bias was moderate [49] and high [50], which limits the usability of the results. Both studies had concerns regarding the risk of bias in the measurement of outcomes. Additional biases were related to confounding [49] and the effect of assignment to intervention [50].

The only organizational intervention identified studied by McGilton et al. [51] did not have clear promotive effects on mental outcomes during COVID-19 pandemic. In the context of long-term care, the impact of facilitated huddles was evaluated using a Bayesian proportional odds model. Most frequently handled topics in the huddles were related to resident care (46%) and staff wellbeing (34%). Results were reported by worker groups (direct care staff, allied care/support staff and management staff). Care workers who attended the huddles reported lower overall moral distress compared to non-attendees (posterior probability = 0.9933). However, management staff attending huddles experienced higher moral distress related to staff turnover and insufficient resident activities. While allied care and support staff reported greater support from the nurse practitioner, management staff attending huddles reported worse physical (posterior probability = 0.04) and mental health (posterior probability = 0.003). Overall, huddles provided mixed results. When interpreting the results, it should be noted that the number of participants in the study was low, and only a few management staff members attended the huddles [51]. The risk of bias was assessed as moderate, with bias detected in areas related to confounding and the measurement of outcomes.

#### Informal caregivers

Eight studies were randomized controlled trials and one quasi-experimental and risk of bias was low in one study [52], moderate/some concerns in four studies [53, 56–58] and high/serious in two studies [54, 59]. One economic evaluation study was assessed as good quality [55]. The main results are summarized in Table 3 and detailed risk of bias assessments in Additional file 2.

The results of support and counselling interventions are promising based on six studies [52–55, 57, 58]. Two studies evaluated supporting interventions with home visits and those seemed most promising [52, 53]. The Home-Based supportive intervention with a waiting list control by Dias et al. [52] observed significant improvements in caregiver mental health (−1.12, 95% CI −2.07 to −0.17) and distress due to behaviour disturbances (−1.96, 95% CI −3.51 to −0.41), with non-significant reductions in perceived burden and functional ability of the carer using mixed effects models [52]. The risk of bias was assessed as low refining to good quality evidence. The study by Araújo et al. [53] found significant interactions between time and group with the intervention group displaying substantial improvements in caregiving skills (*p* < 0.001) and reduced burden (*p* < 0.001) compared to the usual type of care delivered in healthcare units. Mental health also improved slightly in the intervention group compared to the control group, whose condition worsened (*p* = 0.050). The programme did not significantly affect physical health [53]. Risk of bias was assessed as moderate due to performance bias, and potential selective reporting.

Coping interventions delivered through individual therapy sessions or involving both individual and family sessions showed positive results based on two studies [54, 55]. Haley et al. [54] studied a sub-group of participants, caregivers whose care recipient deceased with individual and family sessions. The intervention group showed lower depression scores at one-year post-baseline (estimate = − 1.32, *p* = 0.0138), with a significant linear decrease over time, especially after the care recipient’s death compared to treatment as usual [54]. However, the risk of bias was assessed as high relating to concerns in randomization process and bias in reported results. In addition, another individual coping intervention studied by Livingston et al. [55] showed a mean difference of − 1.80 points (95% CI − 3.29 to − 0.31 points; *p* = 0.02) in anxiety and depression (HADS-T) scores compared to the control group at 8 months. At 24 months, the mean difference was − 2.58 points (95% CI − 4.26 to − 0.90 points; *p* = 0.003), again favouring the intervention. The findings indicate that the intervention effectively decreased anxiety and depression of the carer. The study also assessed cost-effectiveness, and the cost per quality-adjusted life year (QALY) was £6000 in the short term. In the long term, the cost per QALY was £11,200. The authors concluded that the intervention can be considered cost-effective with a willingness-to-pay threshold of £30,000. However, the study found no difference in costs or effectiveness, when measured by QALY [55]. Study quality was assessed as good.

Duncan Davis et al. [56] studied support intervention offered by telephone which managed to achieve a significant reduction in feelings of guilt (F = 5.00, *p* = 0.03) as well as more positive interactions with staff (F = 6.20, *p* = 0.02) compared to no-contact control. However, no significant differences were found in mental health related measures, such as burden, depression and health-related quality of life. In addition, no differences were detected in facility satisfaction, or community resource use [56]. Study was generally well conducted, but some concerns were raised due to lack of pre-defined analysis plan and such, reporting the results.

Ducharme et al. [57, 58] studied supportive group-based intervention for adult-daughter primary caregivers and was evaluated in two publications with different time horizons. In the first article, using a prediction analysis, the intervention group showed more successful outcomes compared to the no programme control group across various measures, including control by self, perceived threat, social support, role overload, reframing, and competence dealing with healthcare staff. Intervention recorded higher success rates (51.1–71.1%) and lower unsuccessful outcomes (28.9–48.9%) compared to the no programme control [57]. The intervention maintained its positive effect at three months follow-up, particularly in dealing with healthcare staff and using reframing strategies [58]. Studies were transparently reported but some concerns were raised in bias of reporting results due to lack of pre-registration or pre-defined analysis plan.

Care management protocol could be an effective intervention in the context of informal care. The study by Montgomery et al. [59] exploring the protocol showed that caregivers in the intervention group experienced significant decreases in identity discrepancy, caregiver burden, depressive symptoms, and intention to place, while the control group experienced the opposite. The intervention group also had lower levels of depressive symptoms compared to the control group [59]. Still, results should be interpreted with caution due to high risk of bias concerning multiple dimensions, such as missing outcomes (high dropout rates), potential measurement bias and reporting the outcomes.

**Table 3:** Effectiveness and economic evaluation results

Study	Mental wellbeing or resilience outcome	Assessment method	Effectiveness/economic evaluation result	Authors conclusion	Risk of bias
**Elderly care workers**					
DeGraves et al. 2023 [49]	Perceived Stress Scale (PSS-10), Patient Health Questionnaire (PHQ-4), Insomnia Severity Index (ISI), Brief Resilience Scale (BRS), Professional Quality of Life Assessment (ProQoL-9)	Mixed-effects linear regression model	Statistically significant improvements were found in perceived stress (PSS-10), psychological distress (PHQ-4), anxiety (PHQ-4), and depression (PHQ-4), insomnia (ISI) and resilience (BRS) in both breathing intervention groups. No statistically significant differences between the assessed groups on any outcome.	Simple coherent breathing intervention was associated with improved scores on mental and physical health scales, including improvements in stress, anxiety, depression, psychological distress, resilience, and insomnia among LTC staff.	Moderate
Hansell et al. 2023 [50]	Perceived Stress Scale (PSS-4), Maslach Burnout Inventory General Survey (MBI-GS), Beck Anxiety Inventory (BAI), Center for Epidemiological Studies- Depression Scale (CESD), Positive and Negative Affect Schedule (PANAS), Insomnia Severity Index (ISI), Pittsburgh Sleep Quality Index (PSQI), Five-Facet Mindfulness Questionnaire (FFMQ)	Mixed models regression	Both interventions (Mindful Awareness Practices (MAPs) and Tai Chi) had significant improvements in outcomes (anxiety, depression, positive affect, perceived stress, levels of insomnia, sleep quality, and improvement in levels of mindfulness). No statistically significant differences between the assessed groups on any outcome.	MAPs were rated and evaluated as more practical to implement in daily life than Tai Chi.	High
Kloos et al. 2019 [46]	General wellbeing by Mental Health Continuum-Short Form (MHC-SF), Maastricht Job Satisfaction Scale for Healthcare (MAS-GZ), Short version of the Utrecht Work Engagement Scale (UWES-S 9)	Linear Mixed Models (LMM)	Positive psychology intervention had no interaction effects on general wellbeing or work engagement. A significant interaction effect found with participants in the intervention condition remaining stable with respect to job satisfaction, and decreasing in controls.	The online multicomponent positive psychology intervention was not effective in improving wellbeing, even for people with low initial wellbeing.	High
McGilton et al. 2023 [51]	Moral Distress in Dementia Care Instrument (adapted 10-item), Supportive Supervisory Scale; Job satisfaction, (How satisfied are you overall with your current job in the LTC home); Overall health (In general, how would you say your health is?) and mental health (In general, how would you say your mental health is?)	Bayesian proportional odds model	Mixed results between worker groups (direct care/managers) in huddle attendees in overall stress and support compared to non-attendees. No evidence of differences in job satisfaction or physical and mental health.	Some evidence that those who attended huddles experienced less moral distress and greater support from the nurse practitioner (NP). NPs are important members of LTC home teams and can be instrumental in implementing evidence-based practices	Moderate
O’Brien et al. 2019 [45]	General Health Questionnaire (GHQ-12)	Repeated measures ANOVAs Follow-up pairwise comparisons Bon- ferroni correction for significance levels.	ACT group reported a significant reduction in mental health symptoms. In contrast, there was no significant change across time for the control group participants. Between- group comparisons at 1 month follow-up indicated that the ACT group reported marginally significant fewer mental health symptoms relative to the control group.	The ACT intervention was associated with fewer days missed due to work-related injury, as well as improved wellbeing, as measured by a reduction in mental health symptoms. A group-based ACT intervention can promote improvements in wellbeing for nurses and nurse aides working in long-term care settings.	Some concerns
Riello et al. 2021 [47]	Self-reported anxiety (GAD-7) and/or post-traumatic symptomatology (IES-R), Resilience (CD-RISC 25), Well-being Index (WHO-5), Perceived Stress Scale (PSS-10)	One-tailed χ2-test	SH + showed no significant difference in symptomology, anxiety, resilience, wellbeing or stress compared to alternative compassionate reading intervention.	No significant difference is found in the rate of self-reported symptoms of anxiety and/or post-traumatic stress between the group receiving the SH + and the group receiving the alternative intervention.	Some concerns
Schoultz et al. 2022 [48]	Perceived Stress Scale (PSS-14), Coping Self-Efficacy Scale	Regression models	Significant differences between PFA and non-PFA groups on coping, safety, calmness, and hopefulness favouring the PFA group (with small effect size). No effect on stress, connectedness, or accomplishment.	Study suggests some benefits to healthcare workers in care home settings undergoing PFA. The presence of increased coping in those who had received PFA gives an indication that it is possible that this was, in some part, an effect of the PFA training.	Serious
**Informal caregivers**					
Araújo et al. 2018 [53]	Skills Scale of Informal Caregivers of Dependent Older People Post-stroke (ECPICID-AVC); Burden (QASCI); Informal caregivers’ health condition (SF-36)	Linear mixed effects models	A significant interaction between time and group in skills, burden and mental health favouring the intervention group. No effect of caregiver’s physical health condition on the intervention programme was found.	The InCARE programme provides positive results in substantial practical skills, as well as a desired burden decrease in informal caregivers 3 months after the implementation of the programme.	Moderate
Duncan Davis et al. 2011 [56]	Caregiver Guilt Questionnaire for Nursing Home Placement; Center for Epidemiology Studies Depression Scale; Burden Interview; Ohio Department of Aging Family Satisfaction Instrument; Health-related quality of life (SF-36), Social support; Negative reactions to care recipient behaviour	Mixed model analysis of variance	FITT-NH had no significant effect on burden, depression, health-related quality of life, facility satisfaction reaction to patient behaviour problems over the 3-month study period compared to non-contact controls. Significant effect on guilt and hassles with staff favouring the intervention group.	Caregivers receiving FITT-NH showed a greater reduction of guilt feelings and more positive interactions with staff compared to those caregivers receiving no additional contact. The intervention was not associated with differential reductions in burden or depressive symptoms.	Some concerns
Dias et al. 2008 [52]	Caregiver mental health (GHQ score), perceived burden (Zarit Burden score), distress due to problem behaviours (NPI-D)	Mixed effects model	Significant improvements in caregiver mental health and perceived burden in home-based support programme compared to waiting list control. Non-significant reductions in behaviour disturbance and functional ability.	Pilot trial shows that a community-based intervention using locally available resources is feasible, acceptable and leads to significant improvements in caregiver mental health and burden of caring.	Low
Ducharme et al. 2005a [57]	Psychological Distress Index; Perceived caregiver-role overload (scale developed by Pearlin et al.); Stress Appraisal Measure (SAM); Carers’ Assessment of Managing Index (CAMI); Competence dealing with healthcare staff (developed by Koren, DeChillo & Friesen)	Prediction analysis	Taking Care of Myself intervention (EG) group obtained successful outcomes predicted for these variables compared to the no programme control group: control by self, perceived threat, social support, role overload, reframing competence dealing with healthcare staff and perceived challenge.	In summary, successful outcomes were more likely only under the EG condition for competence dealing with health care staff and perceived challenge. Other successful outcomes were more likely for both treatment groups (EG and AG) and unsuccessful outcomes were more likely under the CG condition (no intervention) for all these variables.	Some concerns
Ducharme et al. 2005b [58]	Same as Ducharme et al. 2005a	Prediction analysis	Taking Care of Myself intervention (EG) group obtained successful outcomes for three variables (perceived availability of informal/formal social support, Competence dealing with health care staff, Reframing control by self) than those in the no programme control group.	The EG condition was the only condition that maintains two effects, namely effects on caregiver competence dealing with healthcare staff and on use of the coping strategy of reframing.	Some concerns
Haley et al. 2008 [54]	Geriatric Depression Scale	Random effects regression growth curve analyses	The study compared only those participants whose care recipient died. The NYUCI intervention group had lower depression score and a linear decrease in depression was significant across time; this rate of decline was stronger after the care recipient’s death than before compared to no intervention group.	Results suggest that the NYUCI improves caregiver depressive symptoms not only during active caregiving but also during caregivers’ adaptation to bereavement.	High
Livingston et al. 2014 [55]	Hospital Anxiety and Depression Score (HADS-T), EQ-5D, Carer mental health (HSQ score) ICER (cost-effectiveness analysis)	Multilevel mixed models	8 and 24 months: Significant decrease in anxiety and depression score (HADS-T) favouring the START intervention group compared to usual treatment group. No significant differences in costs. No significant differences in QALYs.	The START intervention was clinically effective and cost-effective in the short and longer term.	Good quality (CHEC)
Montgomery et al. 2011 [59]	Identity discrepancy; Caregiver burden; Depressive symptoms; Intention to place	Repeated measures random effects regression	A significant decrease in scores for all outcome measures (identity discrepancy, burden, depressive symptoms, intention to place) in the TCARE intervention group, whereas the scores for treatment as usual controls increased.	The use of the TCARE^®^ protocol, will promote the wellbeing and mental health of caregivers.	High

## Discussion

Our main result is that there is lack of organizational and managerial interventions to promote resilience and mental wellbeing in elderly care. There was only one organizational intervention, huddles during covid, providing mixed results [51]. Organizational interventions in this context can be identified as interventions focusing on changing workplace structures, policies, practices and culture. Leaders play an important role initiating these interventions and thus are considered as change agents in improving working conditions. Better working conditions and the environment lead to enhanced mental wellbeing of the workers [32–34].

A similar shortage of organizational and managerial interventions has been pointed out in the literature in the general health care context [24, 60–62] and in addition sectors other than health care [63]. Previous literature has found that concrete changes in the workplace can be effective, and precisely, flexible work scheduling is effective promoting mental wellbeing and decreasing occupational stress of health care workers [23, 60]. In addition, evidence of job task modification has proven to be effective in promoting mental wellbeing [23].

Among the elderly care worker interventions, only a few demonstrated a positive effect on promoting mental wellbeing outcomes. Notably, psychological group-based intervention called Acceptance and Commitment Therapy (ACT) was found to be beneficial for promoting wellbeing [45]. Other psychological based training interventions were not proven to be effective due to lack of effectiveness [46, 47] or high risk of bias in study quality [48]. Besides the ACT, relaxation interventions with mindfulness or breathing exercises seemed to have a beneficial effect on wellbeing [49, 50]. However, due to lack of pure comparators in both studies and other limitations in study quality, the evidence of relaxation interventions should be treated with caution. Previous literature has also found that individual targeted interventions that used structured therapeutic methods (such as ACT, CBT), and focusing on mindfulness, relaxation and coping skills, can have positive effects in improving mental health outcomes, such as burnout, stress, depression, emotional exhaustion, and general wellbeing among health care workers [24, 64, 65]. Still, further research is needed that expands the range of factors and outcomes examined to include positive concepts of wellbeing, vitality and sustainability [62], especially in the field of health and social care.

In contrast, a higher proportion of informal carer interventions showed positive outcomes related to mental wellbeing. Effective strategies included support and counselling, practical skills training, awareness and knowledge improvement, individual coping therapy, and computer-assisted care management protocols [52–55, 57–59]. Nevertheless, the quality of studies was heterogeneous. Supporting interventions provided at home has the strongest evidence of being effective [52, 53]. In addition, evidence of individual therapy sessions, and group-based support intervention is moderate and can be considered as effective strategies [55, 57, 58]. Instead, the combination of individual and family sessions and care management protocols showed some results of effectiveness, but study quality was weak in both studies and thus, evidence can be considered as weak [54, 59]. Telephone based support found no effect on mental health related outcomes [56]. Literature has found desirable effect on informal caregiver´s psychological distress and depression with wide range of interventions; psychoeducation, psychosocial, multicomponent, cognitive behavioral therapy, and mindfulness-based and support group interventions can promote mental health of the caregiver [66, 67]. Interventions included in this review underscore the importance of tailored support programmes for caregivers, to enhance the coping of caregivers.

This review found only one economic evaluation study of a coping intervention to support carers of people with dementia [55]. Authors concluded that the intervention was cost-effective, hence they did not find significant differences in costs or effectiveness measured with QALYs between the intervention and comparator. Also previous economic evaluation studies stress the importance of multi-component programmes—encompassing self-care, communication, skill development, awareness-raising, knowledge of relevant services, and psychosocial elements [38]. Multi-component interventions are more likely to be cost-effective when customized to the specific circumstances of carers and delivered in person [38]. The fact that we found only one economic evaluation study reveals the significant gap in the literature, as understanding the cost-effectiveness of interventions is vital for making informed decisions about resource allocation and for advocating for the implementation of successful interventions. The lack of workforce and the increase in mentally exhausted workers in the health and social care sector highlights the importance of economically sustainable solutions to keep important workers in their jobs and mentally in good shape. Better mental wellbeing reflects better outcomes in the workplace and from the economic perspective, potentially reduced sick leaves and absenteeism and thus, economic evaluation studies concerning promotion of mental wellbeing could concentrate to explore these associations.

Our review also reveals that the research is mainly individual oriented and focused on mental ill health and negative outcomes such as depression or burnout with comparatively little evidence on the impact on positive mental wellbeing and resilience. The concept resilience combines organizational orientation and on individual level focuses on positive mental health. Resilience can be defined as an individual trait but also a characteristic of systems and organizations, the collective ability to modify work practices [26]. Worker resilience can be influenced by leadership styles and organizational cultures [34]. Health interventions are mostly directed at individuals [60], but organizational interventions can have more sustainable effects than individual targeted interventions supporting worker wellbeing [68, 69]. There is clearly a need for organizational and management interventions to promote resilience and mental wellbeing in elderly care.

The strength of this review was its coherent search across multiple databases. In addition, well planned and demonstrated work processes, such as reporting, calibration of the screening phase, and working in pairs improved the quality of this review. Our multidisciplinary research team also brought together different research fields and enriched the review process with relevant background discussions about the topic and understanding the phenomenon from different angles. Our review also has some limitations. There is a possibility that we missed some relevant studies due to strict inclusion and exclusion criteria, and we did not search for unpublished studies and other grey literature. We only accepted studies published in peer-reviewed journals and written in English. Another limitation of this study was that unfortunately, we were not able to transform the outcomes into standardized effectiveness measures, which was our original intention. We could not complete it due to the variety of measures used estimating the effectiveness.

### Research implications

The lack of good quality research concerning organizational and managerial interventions can be due to multiple reasons. There may appear real-world barriers in implementing new interventions and practices, such as lack of resources, poor participant commitment, insufficient management support or lack of prioritization of new interventions [70–72]. In addition, organizational characteristics, such as rigid hierarchies, the uniqueness of each working units, or resistance to change may hinder the implementation of interventions [70, 72]. Organizational interventions often suffer from definitional ambiguity, making them hard to study or implement consistently compared to individual level interventions, which may be more easily studied [72]. However, as health and social care challenges evolve, ongoing evaluation and adaptation of new programmes are essential to meet the diverse needs of workers in the care giving field effectively. To successfully safeguard the mental health of these workers, interventions should be comprehensive, tailored and multi-dimensional and be applied at various levels [62, 63].

Mental health and wellbeing concepts are evaluated using various measures, which complicates the comparison of intervention effectiveness across studies [62]. Many studies included in this review did not have a protocol published before the trial, and the studies did not specify the primary outcome which hindered summarizing the results. Due to the variety of outcomes (and interventions), we cannot make a strong summary of which outcomes these interventions positively affected. Same observations have been made in other reviews, and future research would benefit for harmonizing the measured outcomes [66] and defining a core outcome set for mental wellbeing and resilience.

We conclude that research needs to innovate holistic interventions for workers and informal caregivers and not only focus on the individual approach. The co-creation of interventions presents a promising approach for developing contextually appropriate and effective solutions by actively engaging stakeholders in the processes of problem identification, as well as in the design and selection of the most suitable intervention strategies [73]. The available frameworks offer a structured and practice-oriented approach for researchers and stakeholders to systematically design and implement evaluations of co-creation processes and the methodologies employed therein [74, 75]. Co-creation has been applied to research in home care settings, too. Home care workers, together with researchers, acted as identifiers of older home care recipients’ unmet social needs [76].

In addition, allocation of funding to enable longitudinal intervention studies with proper follow-up should be prioritized. Another way to provide valuable insights would be examining interventions that focus on patients and care recipients and while changing working practices can also promote the wellbeing of workers and carers e.g.[77].

### Practical implications

Our systematic review shows that supporting informal caregivers with counselling, practical skills training, awareness and knowledge improvement and coping therapy promotes their mental wellbeing and resilience. These multicomponent interventions including psychotherapy, support, and educational components can be implemented in various service settings and provision modes, preferrable through home visits or individual therapy and family sessions.

For workers, in stressful situations group therapy sessions or relaxation techniques might help. However, they do not help to change practices at work. There is a clear shortage of research that would support manager’s role in promotion of mental wellbeing at work. Thus, we encourage leaders in organizations to co-create interventions with researchers that empower organizations and leaders to reframe and improve working conditions together with workers.

## Conclusion

Tailored support and coping interventions for informal carers enhance their mental wellbeing. This review indicates that interventions for workers are targeted at individuals yet there is notable scarcity of evidence on managerial or organizational interventions. Addressing this gap is crucial for developing strategies for leaders together with workers to improve the resilience of organizations and thus also the mental wellbeing of workers. While it is relatively straightforward to identify problems within the caregiving field, finding evidence-based solutions remains.

## Supplementary Information

Supplementary Material 1.

Supplementary Material 2. [78]

## Data Availability

No new data were created or analyzed in this study. The datasets formed and summarized during the current study are available from the corresponding author (AKV) on reasonable request.

## Abbreviations

ACT: Acceptance and Commitment Therapy
AD: Alzheimer’s Disease
CBT: Cognitive Behavioural Therapy
CHEC: Consensus on Health Economic Criteria
cRCT: Cluster Randomized Controlled Trial
FITT-NH: Telephone Tracking-Nursing Home
ICER: Incremental Cost-Effectiveness Ratio
MAP: Mindfulness Awareness Practice
NYUCI: The New York University Caregiver Intervention
PFA: Psychological First Aid
PRISMA: Preferred Reporting Items for Systematic Review and Meta-Analyses
QALY: Quality-Adjusted Life Year
RCT: Randomized Controlled Trial
ROBINS-I: Risk Of Bias In Non-Randomized Studies
SH: Self-help
START: The Strategies for Relatives
TCARE: Tailored Caregiver Assessment and Referral protocol
UK: United Kingdom
US: United States
WEMWBS: Warwick-Edinburgh Mental Wellbeing Scale
WHO: World Health Organization
