# Supplementary material for: The effectiveness and economic evidence of organizational and management interventions to promote mental wellbeing and resilience in elderly care workers and informal caregivers – a systematic review
Source: BMC Health Serv Res. 2025 Oct 10;25:1345. doi: 10.1186/s12913-025-13372-7 (PMC12512583; doi:10.1186/s12913-025-13372-7)
Supplement: Supplementary file 1 — Supplementary Material 1. [file 12913_2025_13372_MOESM1_ESM.docx]

**Additional file 1 – Search strategy**

**Basic Search Strategy**

("residential care" OR "home care" OR "nursing home*" OR "elder* care" OR "care for the elderly" OR "long term care" OR “Aged care” OR "residential facilit*" OR "nursing care facilit*" OR "old age home" OR "assisted living facilit*" OR "home for the aged" OR "housing for the elderly" OR "residential aged" OR "informal care" OR "Home Health Care")

AND (employee* OR staff OR personnel OR worker* OR nurse* OR physician* OR "social worker*" OR professional OR carer OR caregiver)

AND (organisation* OR organization* OR strateg* OR administrat* OR system OR systems OR facilit* OR manage* OR leader* OR workplace OR superior OR supervisor OR head)

AND (resilien* OR "mental health" OR "mental well-being" OR "mental wellbeing" OR "psychological well-being" OR "psychological wellbeing" OR hardiness or responsiveness OR adapt*)

AND (effect* OR effic* OR evaluat* OR impact* OR promot* OR "economic evaluation" OR "economic assess*" OR "cost effectiveness" OR "cost utility" OR "cost benefit" OR "cost minimization" OR "cost consequence")

**PubMed (n= 1196)**

Search: ("residential care"[Title/Abstract] OR "home care"[Title/Abstract] OR "nursing home*"[Title/Abstract] OR "elder* care"[Title/Abstract] OR "care for the elderly"[Title/Abstract] OR "long term care"[Title/Abstract] OR "Aged care"[Title/Abstract] OR "residential facilit*"[Title/Abstract] OR "nursing care facilit*"[Title/Abstract] OR "old age home"[Title/Abstract] OR "assisted living facilit*"[Title/Abstract] OR "home for the aged"[Title/Abstract] OR "housing for the elderly"[Title/Abstract] OR "residential aged"[Title/Abstract] OR "informal care"[Title/Abstract] OR "Home Health Care"[Title/Abstract]) AND (employee*[Title/Abstract] OR staff[Title/Abstract] OR personnel[Title/Abstract] OR worker*[Title/Abstract] OR nurse*[Title/Abstract] OR physician*[Title/Abstract] OR "social worker*"[Title/Abstract] OR professional[Title/Abstract] OR carer[Title/Abstract] OR caregiver[Title/Abstract]) AND (organisation*[Title/Abstract] OR organization*[Title/Abstract] OR strateg*[Title/Abstract] OR administrat*[Title/Abstract] OR system[Title/Abstract] OR systems[Title/Abstract] OR facilit*[Title/Abstract] OR manage*[Title/Abstract] OR leader*[Title/Abstract] OR workplace[Title/Abstract] OR superior[Title/Abstract] OR supervisor[Title/Abstract] OR head[Title/Abstract]) AND (resilien*[Title/Abstract] OR "mental health"[Title/Abstract] OR "mental well-being"[Title/Abstract] OR "mental wellbeing"[Title/Abstract] OR "psychological well-being"[Title/Abstract] OR "psychological wellbeing"[Title/Abstract] OR hardiness[Title/Abstract] OR responsiveness[Title/Abstract] OR adapt*[Title/Abstract]) AND (effect*[Title/Abstract] OR effic*[Title/Abstract] OR evaluat*[Title/Abstract] OR impact*[Title/Abstract] OR promot*[Title/Abstract] OR "economic evaluation"[Title/Abstract] OR "economic assess*"[Title/Abstract] OR "cost effectiveness"[Title/Abstract] OR "cost utility"[Title/Abstract] OR "cost benefit"[Title/Abstract] OR "cost minimization"[Title/Abstract] OR "cost consequence"[Title/Abstract]) Filters: from 2000 – 2024

**EBSCO (n=1805)**

((TI ("residential care" OR "home care" OR "nursing home*" OR "elder* care" OR "care for the elderly" OR "long term care" OR “Aged care” OR "residential facilit*" OR "nursing care facilit*" OR "old age home" OR "assisted living facilit*" OR "home for the aged" OR "housing for the elderly" OR "residential aged" OR "informal care" OR "Home Health Care"))

OR (AB ("residential care" OR "home care" OR "nursing home*" OR "elder* care" OR "care for the elderly" OR "long term care" OR “Aged care” OR "residential facilit*" OR "nursing care facilit*" OR "old age home" OR "assisted living facilit*" OR "home for the aged" OR "housing for the elderly" OR "residential aged" OR "informal care" OR "Home Health Care")))

AND

((TI (employee* OR staff OR personnel OR worker* OR nurse* OR physician* OR "social worker*" OR professional OR carer OR caregiver))

OR (AB (employee* OR staff OR personnel OR worker* OR nurse* OR physician* OR "social worker*" OR professional OR carer OR caregiver)))

AND

((TI (organisation* OR organization* OR strateg* OR administrat* OR system OR systems OR facilit* OR manage* OR leader* OR workplace OR superior OR supervisor OR head))

OR (AB (organisation* OR organization* OR strateg* OR administrat* OR system OR systems OR facilit* OR manage* OR leader* OR workplace OR superior OR supervisor OR head)))

AND

((TI (resilien* OR "mental health" OR "mental well-being" OR "mental wellbeing" OR "psychological well-being" OR "psychological wellbeing" OR hardiness or responsiveness OR adapt*))

OR (AB (resilien* OR "mental health" OR "mental well-being" OR "mental wellbeing" OR "psychological well-being" OR "psychological wellbeing" OR hardiness or responsiveness OR adapt*)))

AND

((TI (effect* OR effic* OR evaluat* OR impact* OR promot* OR "economic evaluation" OR "economic assess*" OR "cost effectiveness" OR "cost utility" OR "cost benefit" OR "cost minimization" OR "cost consequence"))

OR (AB (effect* OR effic* OR evaluat* OR impact* OR promot* OR "economic evaluation" OR "economic assess*" OR "cost effectiveness" OR "cost utility" OR "cost benefit" OR "cost minimization" OR "cost consequence")))

Limitation: Publication Date: 20000101-20241231.

**Web of Science (n=1288)**

((TI= ("residential care" OR "home care" OR "nursing home*" OR "elder* care" OR "care for the elderly" OR "long term care" OR “Aged care” OR "residential facilit*" OR "nursing care facilit*" OR "old age home" OR "assisted living facilit*" OR "home for the aged" OR "housing for the elderly" OR "residential aged" OR "informal care" OR "Home Health Care"))

OR (AB= ("residential care" OR "home care" OR "nursing home*" OR "elder* care" OR "care for the elderly" OR "long term care" OR “Aged care” OR "residential facilit*" OR "nursing care facilit*" OR "old age home" OR "assisted living facilit*" OR "home for the aged" OR "housing for the elderly" OR "residential aged" OR "informal care" OR "Home Health Care")))

AND

((TI= (employee* OR staff OR personnel OR worker* OR nurse* OR physician* OR "social worker*" OR professional OR carer OR caregiver))

OR (AB= (employee* OR staff OR personnel OR worker* OR nurse* OR physician* OR "social worker*" OR professional OR carer OR caregiver)))

AND

((TI= (organisation* OR organization* OR strateg* OR administrat* OR system OR systems OR facilit* OR manage* OR leader* OR workplace OR superior OR supervisor OR head))

OR (AB= (organisation* OR organization* OR strateg* OR administrat* OR system OR systems OR facilit* OR manage* OR leader* OR workplace OR superior OR supervisor OR head)))

AND

((TI= (resilien* OR "mental health" OR "mental well-being" OR "mental wellbeing" OR "psychological well-being" OR "psychological wellbeing" OR hardiness or responsiveness OR adapt*))

OR (AB= (resilien* OR "mental health" OR "mental well-being" OR "mental wellbeing" OR "psychological well-being" OR "psychological wellbeing" OR hardiness or responsiveness OR adapt*)))

AND

((TI= (effect* OR effic* OR evaluat* OR impact* OR promot* OR "economic evaluation" OR "economic assess*" OR "cost effectiveness" OR "cost utility" OR "cost benefit" OR "cost minimization" OR "cost consequence"))

OR (AB= (effect* OR effic* OR evaluat* OR impact* OR promot* OR "economic evaluation" OR "economic assess*" OR "cost effectiveness" OR "cost utility" OR "cost benefit" OR "cost minimization" OR "cost consequence")))

**SCOPUS (n=1626)**

TITLE-ABS ( ( "residential care" OR "home care" OR "nursing home*" OR "elder* care" OR "care for the elderly" OR "long term care" OR "Aged care" OR "residential facilit*" OR "nursing care facilit*" OR "old age home" OR "assisted living facilit*" OR "home for the aged" OR "housing for the elderly" OR "residential aged" OR "informal care" OR "Home Health Care" ) AND ( employee* OR staff OR personnel OR worker* OR nurse* OR physician* OR "social worker*" OR professional OR carer OR caregiver ) AND ( organisation* OR organization* OR strateg* OR administrat* OR system OR systems OR facilit* OR manage* OR leader* OR workplace OR superior OR supervisor OR head ) AND ( resilien* OR "mental health" OR "mental well-being" OR "mental wellbeing" OR "psychological well-being" OR "psychological wellbeing" OR hardiness OR responsiveness OR adapt* ) AND ( effect* OR effic* OR evaluat* OR impact* OR promot* OR "economic evaluation" OR "economic assess*" OR "cost effectiveness" OR "cost utility" OR "cost benefit" OR "cost minimization" OR "cost consequence" ) ) AND PUBYEAR > 1999 AND PUBYEAR < 2025
