# Supplementary material for: The effectiveness and economic evidence of organizational and management interventions to promote mental wellbeing and resilience in elderly care workers and informal caregivers – a systematic review
Source: BMC Health Serv Res. 2025 Oct 10;25:1345. doi: 10.1186/s12913-025-13372-7 (PMC12512583; doi:10.1186/s12913-025-13372-7)
Supplement: Supplementary file 2 — Supplementary Material 2. [78] [file 12913_2025_13372_MOESM2_ESM.docx]

**Additional file 2 – Quality Assessments**

Quality assessment “traffic light” plots are produced with robvis webtool [78].

Quality assessments of RCT studies using RoB2 tool.


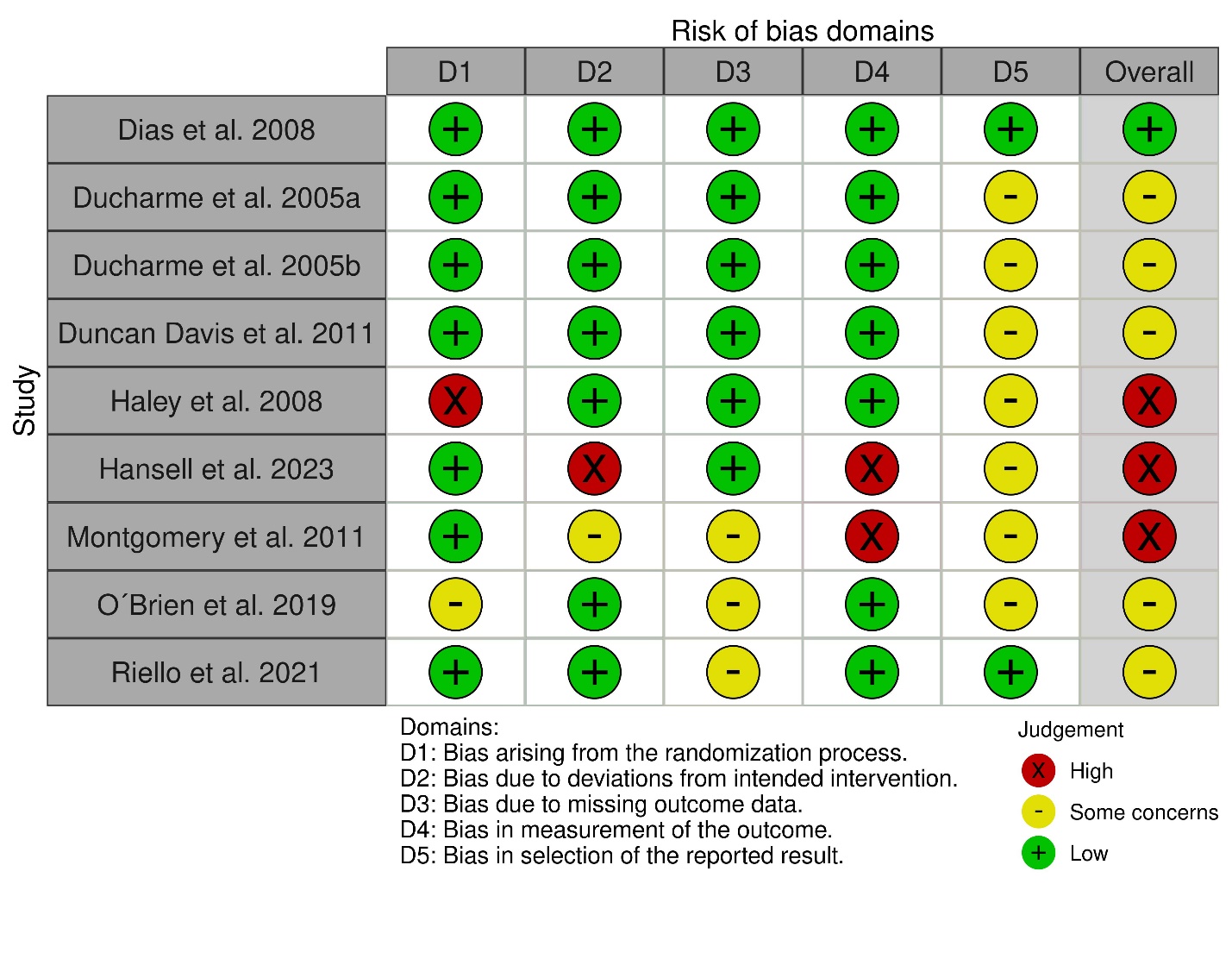


Quality assessment of cRCT study using RoB2 tool for cRCT.


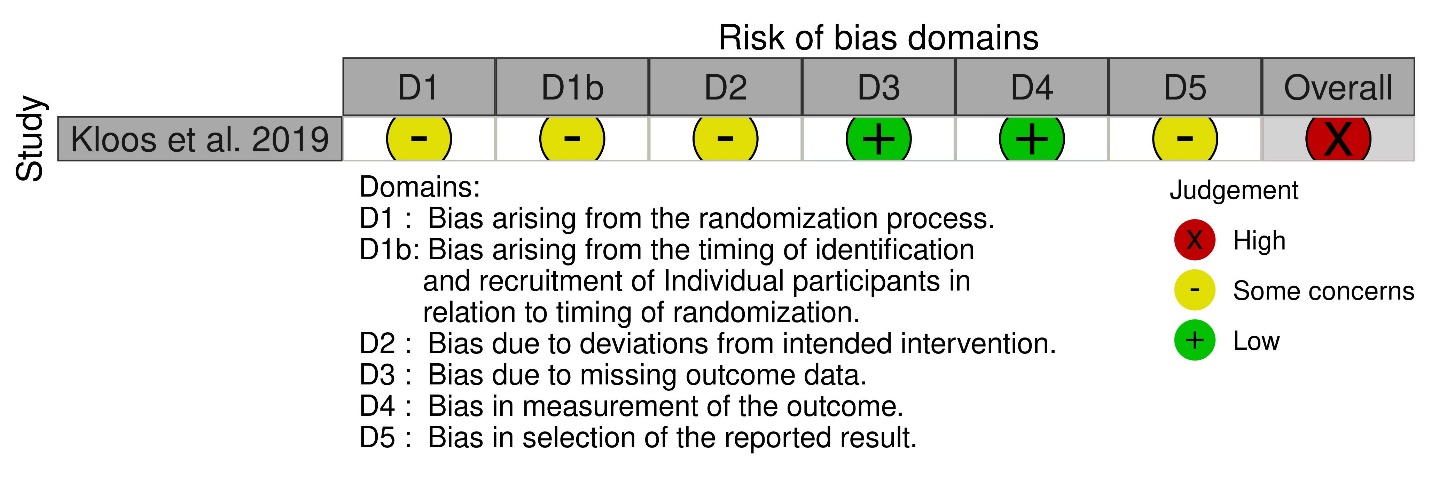


Quality assessment of non-randomized studies using ROBINS-I tool.


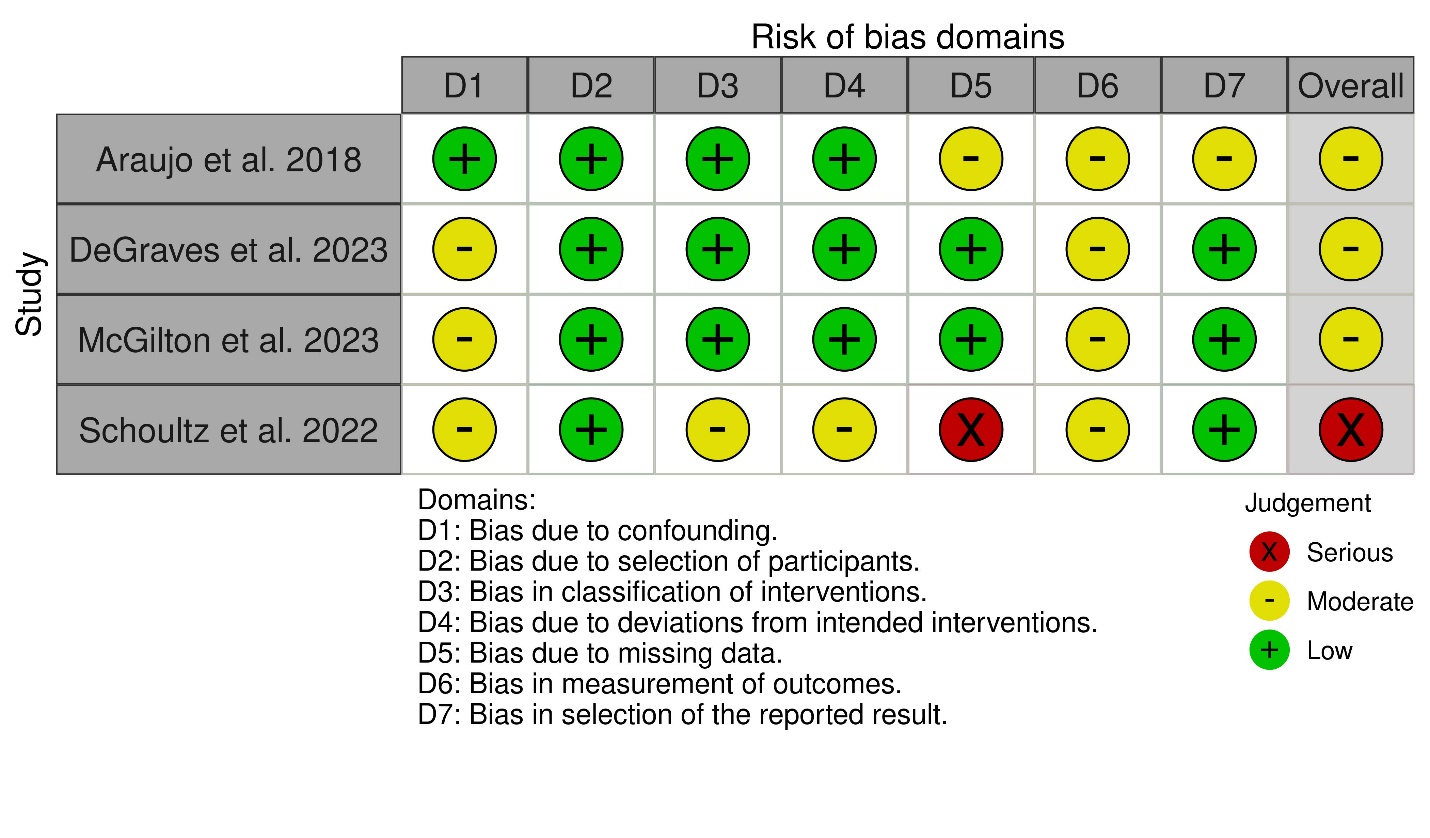


Quality assessment of economic evaluation study using CHEC-list.
